# Profiling DNA Methylation Based on Next-Generation Sequencing Approaches: New Insights and Clinical Applications

**DOI:** 10.3390/genes9090429

**Published:** 2018-08-23

**Authors:** Daniela Barros-Silva, C. Joana Marques, Rui Henrique, Carmen Jerónimo

**Affiliations:** 1 Cancer Biology and Epigenetics Group, IPO Porto Research Center (CI-IPOP), Portuguese Oncology Institute of Porto (IPO Porto), Rua António Bernardino Almeida, 4200-072 Porto, Portugal; daniela.barros.silva94@gmail.com (D.B.-S.); rmhenrique@icbas.up.pt (R.H.); 2 Genetics, Department of Pathology, Faculty of Medicine, University of Porto, 4200-319 Porto, Portugal; cmarques@med.up.pt; 3 I3S—Instituto de Investigação e Inovação em Saúde, Universidade do Porto, 4200-135 Porto, Portugal; 4 Department of Pathology, Portuguese Oncology Institute of Porto (IPO Porto), 4200-072 Porto, Portugal; 5 Department of Pathology and Molecular Immunology, Institute of Biomedical Sciences Abel Salazar (ICBAS)—University of Porto, 4050-313 Porto, Portugal

**Keywords:** epigenomics, DNA methylation, next-generation sequencing, single-cell resolution

## Abstract

DNA methylation is an epigenetic modification that plays a pivotal role in regulating gene expression and, consequently, influences a wide variety of biological processes and diseases. The advances in next-generation sequencing technologies allow for genome-wide profiling of methyl marks both at a single-nucleotide and at a single-cell resolution. These profiling approaches vary in many aspects, such as DNA input, resolution, coverage, and bioinformatics analysis. Thus, the selection of the most feasible method according with the project’s purpose requires in-depth knowledge of those techniques. Currently, high-throughput sequencing techniques are intensively used in epigenomics profiling, which ultimately aims to find novel biomarkers for detection, diagnosis prognosis, and prediction of response to therapy, as well as to discover new targets for personalized treatments. Here, we present, in brief, a portrayal of next-generation sequencing methodologies’ evolution for profiling DNA methylation, highlighting its potential for translational medicine and presenting significant findings in several diseases.

## 1. Introduction

DNA methylation patterns’ changes have been widely studied and constitute the most well understood epigenetic modification. The addition of a methyl group at the 5′ position of the cytosine residue proved to be an essential mechanism in both gene expression and chromatin structure regulation [1]. The functional presence of 5mC (5-methylcytosine) in gene promoters is generally associated with transcriptional repression due to structural chromatin alterations, while the absence is linked with transcriptional activity. Gene body methylation also plays a major role in repetitive DNA elements’ silencing and alternative splicing [2,3]. DNA methylation has been associated with several biological processes such as genomic imprinting, transposon inactivation, stem cell differentiation, transcription repression, and inflammation [4]. DNA methylation profiles can also be inherited through cell division and sometimes through generations [5]. Since methyl marks play a very relevant role in both physiologic and pathologic conditions, the significance of profiling DNA methylation to answer biological questions has been highlighted. Moreover, uncovering of DNA methylation genomic regions is appealing to translational research because methyl sites are modifiable by pharmacologic intervention [6] and are easy to measure in liquid biopsies through cost-effective methods [7]. However, efforts to examine epigenetic modifications in health and disease have been hindered by the lack of high-throughput and quantitatively accurate approaches, until recently.

Over the last years, numerous methods were developed to map 5mC providing a genome-wide coverage of DNA methylation changes. This improvement in DNA methylation analysis techniques was only possible due to the advances of various profiling approaches, both experimental and computational [8,9,10]. Epigenetics was one of the first molecular fields capitalizing next-generation sequencing (NGS) methodologies to its favor, providing a comprehensive and unbiased view of the epigenome and also releasing researchers from content-limited microarray platforms [11]. Next-generation sequencing technology has brought unprecedented advancement to epigenomic research, particularly in DNA methylation landscape. Compared to array-based technologies, NGS made possible deep sequencing in a short time (from one to few days), producing billions of short DNA samples known as reads (ranging from 50–400 nucleotides) and providing better coverage of all possible methylation sites in the human genome [12]. The arrival of NGS technologies allows for each of the three billion bases in the human genome to be sequenced multiple times, enabling high depth reading to deliver accurate data and insight into unexpected DNA variations [13]. Thanks to massively parallel sequencing it is now possible to recognize methylation status on a large scale and at a single-base resolution [9].

A complete characterization of the methylome and the dynamic changes that occur within may serve as an accurate diagnostic, prognostic, and predictive tools. This mini-review aims to provide a brief overview of NGS tools that might be used in translational medical research, discussing the main advantages and limitations of those technologies and making considerations about the advantages and limitations of each method.

## 2. DNA Methylation Profiling

Pyrosequencing, methylation-specific polymerase chain reaction (PCR), and direct Sanger sequencing have been the most widely used methods for analysis of targeted regions, such as a promoter region of a single gene or a CpG (Cytosine-phosphate-Guanine) island. Although highly useful, the limitations of these techniques include low quantitative accuracy, short read length, and low sample throughput.

A plethora of new methods are currently used to investigate 5mC epigenetic landscape in DNA. Microarray hybridization was one of the first technologies escalating the DNA methylation studies to a genome-wide level. Notably, the methylation arrays are cost-effective tools which do not require large amounts of input DNA, enabling simultaneous analysis of several samples. However, the coverage is highly dependent on the array design [14]. Next-generation sequencing platforms are now emerging, allowing for massive analysis of the methylation status of almost every CpG site and construction of DNA methylation’s genomic maps at a single base resolution [15]. Nevertheless, most of the approaches to 5mC analysis still have the restrictions of being density-biased, deficient in robustness and consistency, or incapable of analyzing 5mC specifically [16]. Hence, for DNA methylation studies involving clinical research, the combination of next-generation sequencing and methylation arrays may constitute a powerful approach as discovery and screening tools, respectively [9].

**Figure 1:**
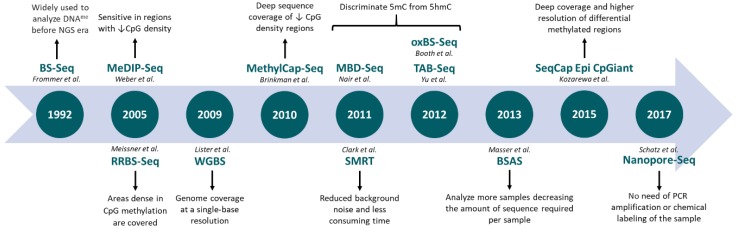
Evolution of next-generation sequencing-based techniques applied to DNA methylation profiling. BS-Seq: bisulfite sequencing; MeDIP-Seq: methylated DNA immunoprecipitation sequencing; RRBS-Seq: reduced representation bisulfite sequencing; WGBS: whole genome bisulfite sequencing; MethylCap-Seq: methylation capture sequencing; MBD-Seq: methyl-CpG binding domain sequencing; oxBS.Seq: oxidative bisulfite sequencing; TAB-Seq: TET-associated bisulfite sequencing; BSAS: bisulfite amplicon sequencing.

A detailed characterization of the most commonly used genome-wide techniques is depicted in Table 1. An historic overview of NGS-based methods applied to epigenomics is shown in Figure 1.

### 2.1. Affinity Enrichment-Based Methods

This strategy uses antibodies (methylated DNA immunoprecipitation or MeDIP-Seq) and methylated-CpG binding proteins (MBD-Seq) to pull down the genomic regions that are methylated for sequencing. The unmethylated genomic fraction is washed away and the methylation-enriched portion is then collected and sequenced [17,18]. Although enrichment techniques are biased towards hypermethylated areas, which are preferentially or more effectively captures, they are particularly useful for characterizing enriched CpG regions (CpG islands and promoter regions).

The main limitations of these approaches are the limited quantification across regions and lack of base-specific data analysis, which ultimately diminishes the insight that could be gathered from them. Thus, these affinity-based methodologies require substantial experimental and bioinformatics adjustments [9]. Sequence data are more commonly analyzed using count-based models, but this statistical method should be avoided in large group samples’ analysis since the biological variability is ignored and more false positives are generated. In this context, a beta-binomial model works more favorably, because it is flexible enough to capture both the technical (coverage) and biological (methylation level) variability [19,20].

### 2.2. Restriction Enzymes-Based Methods

Restriction-enzyme-based approaches take advantage of restriction enzymes (MspI) that are able to cleave the recognition sequence at the site of DNA methylation (CCGG motifs), thus, 5mC can be identified in selected sequences [21]. This is the most time and cost-effective sequencing method and require minute amounts of DNA [22]. However, only lower weight fragments, between 40–220 bp, are suitable for sequencing, despite a wide range of lengths resulting from digestion. The primary limitation to this method is the inability to tune coverage regions of interest due to the dependence on the restriction sites location in the genome, leading to a lack of applicability in genes with sparse CCGG motifs [23].

### 2.3. Bisulfite Conversion-Based Methods

In bisulfite sequencing (BS-Seq), denatured DNA is subjected to bisulfite treatment during which the unmodified cytosine is converted to uracil, but a methylated cytosine remains unchanged, thus allowing base resolution detection of cytosine methylation [24,25]. The bisulfite treatment makes methylation sequencing data processing challenging because of C → T conversion. Thus, it is important to bear in mind that bisulfite sequence reads are not complementary to the reference genome and, therefore, special alignment tools are usually required [19]. In this particular case another level of complexity is added because every given thymine could either be a genuine genomic thymine or a converted unmethylated cytosine, rendering conventional alignment tools, such as Bowtie or BWA (Burrows-wheeler aligner), unsuitable [26]. However, several new computational tools have been developed to address this issue, namely BSMAP [27] and Segemehl [28], which enumerate all C to T combinations in the read, or BISMARK [29] and BS-Seeker [30], which convert all C to T in both sequenced reads and genome reference prior to alignment.

Whole genome bisulfite sequencing (WGBS) is the most informative but also the most expensive base resolution technology since whole genome is targeted by this method. For WGBS, genomic DNA libraries are created and subsequently bisulfite converted, sequenced, and mapped back to the reference genome. Although BS-seq is the most direct assay and displays the highest resolution for methylation detection, this methodology is only used for seeking specific questions in which comprehensive DNA methylation profile is required [31].

Over the last years, numerous WGBS studies demonstrated that the majority of CpG sites are equally methylated and only a small portion of these regions depicts variable DNA methylation, the so-called differentially methylated regions (DMR). Thus, development of new sequencing approaches, more useful for studies testing specific regions of interest and capable of focus only in DMR are needed to efficiently validate putative candidate loci across a large number of samples. In this context, Masser et al. developed an approach, termed bisulfite amplicon sequencing (BSAS), for hypothesis driven and focused absolute DNA methylation analysis [32]. With the onset of NGS platforms it was possible to sequence multiple samples in parallel in one run using multiplexed amplicon-based NGS [33]. This new methodology is highly sensitive and integrates DNA barcoding into the library construction process, so that many samples (from 96 up to 384 different samples) can be pooled to fully use NGS capacity [34].

### 2.4. Oxidative Bisulfite Conversion-Based Methods

One of the most recent developments in epigenomic profiling was the discovery of alternative cytosine modifications with relevant functional biologic roles, including 5-hydroxymethylcytosine (5hmC), 5-carboxylcytosine (5caC), and 5-formylcytosine (5fC), Ito et al. [35], which are postulated to be involved in the process of DNA demethylation [36]. Conventional bisulfite sequencing is not able to distinguish between 5mC and 5hmC and the methylation profile resulting from this methodology includes the sum of both epi-marks [37]. Indeed, when specific techniques were applied for hydroxymethylation discrimination, a quarter of what was previously assumed as methylation was, in fact, hydroxymethylation [38]. Thus, the development of new methods for DNA profiling based on NGS that discriminate between methylation and hydroxymethylation was imperative. A modified bisulfite sequencing approach—oxidative bisulfite sequencing (Ox-BS)—includes a selective oxidative step that deprotects hydroxymethylation and converts 5hmC to 5fC, which, after bisulfite treatment, becomes a uracil [39,40]. Then a subtractive approach between bisulfite sequencing (BS) and Ox-BS libraries allows for methylation and hydroxymethylation quantification at single-base resolution. The main drawbacks of Ox-BS are the oxidative degradation of DNA and longer bisulfite treatment required for complete 5fC deamination [41].

An alternative method for 5hmC detection is Tet-assisted bisulfite sequencing (TAB-Seq), which might be used for whole genome or locus-specific sequencing [42]. This technique encompasses β-glucosyltransderase-mediated protection of 5hmC followed by Tet1-mediated oxidation of 5mC to 5caC. Then, bisulfite treatment and subsequent PCR amplification take place and 5caC, as well as cytosine, are converted to thymine, whereas 5hmC reads as cytosine [43]. Compared with OxBS-Seq, TAB-Seq directly reads 5hmC and the treatment methodology incurs less DNA damage. Nevertheless, TAB-Seq entails highly active Tet protein to enable efficient conversion of 5mC into 5caC [44].

### 2.5. Capture-Based Methods

The ability to capture and sequence large contiguous DNA fragments represents a significant advance towards comprehensive characterization of complex genomic regions. Capture-based DNA sequencing is advantageous not only because it is more cost-effective, as it facilitates higher sample throughput than whole genome sequencing, but also because it improves accuracy by optimizing the read depth coverage and by reducing the complexity of the DNA to be sequenced [45].

Several studies have highlighted the importance of differential methylation outside CpG islands as disease-associated determinants [46,47,48]. Thus, a new methylation-capture method—MethylCap-seq—was developed aiming at deep sequence coverage of lower CpG density regions. This technique is based on DNA methylation capture with MBD (methyl-CpG binding) domain of MeCP2 (methyl-CpG binding protein 2), which is advantageous for a highly controlled and stepwise elution and stratification of methylated DNA fragments according to methyl-CpG density. Currently, MethylCap-seq has been efficiently robotized enabling high reproducibility among numerous samples [18].

Another common bisulfite methylation sequencing method is SeqCap Epi CpGiant which is based on capture of bisulfite-converted DNA. This is also a target-enrichment protocol for genomic regions where methylation is known to impact gene regulation. It allows for sequencing of pre-selected regions with a genome coverage of approximately 80 million bases and 5 million CpG sites [19].

### 2.6. Third-Generation Sequencing

This recent technology allows for DNA modifications analysis without previous chemical conversion. Although conversion-based sequencing is currently the most used NGS methodology, this has some flaws, including difficulties in conversion efficiency control and in accurate alignment of altered sequences to their reference genome. Clark et al. [49] established an alternative approach, consisting on single-molecule real-time (SMRT) DNA sequencing to recognize modified DNA bases in the DNA template directly. This new methodology is based on changes in the kinetics of DNA polymerase (stretches of fluorescent signals represent the dynamics of DNA polymerization) during the occurrence of the modified bases [49,50]. In the same line, another method based on nanopore sequencers allows for direct read of different modifications on DNA bases. Nanopore sequencing uses pores though which nucleic acid strands are pulled, and the consequent ionic pattern reveals the nucleotide sequence, including modifications [51,52]. Although these technologies are still in the development phase, they seem promising for future methylome profiling analysis.

## 3. New Insights from Next-Generation Sequencing on Methylome Analysis: Strengths and Weaknesses

Next-generation sequencing revolutionized the methylome analysis contributing with a variety of new methods which expanded knowledge and characterization of differentially methylated DNA regions (Table 2). Especially in clinical research, DNA methylation status assessment through NGS in clinical samples might provide relevant information on diagnosis and prognosis. Furthermore, pharmacoepigenomics is another promising clinical epigenetic field in which the use of NGS might be of great importance, since the methylation status at specific candidate gene promoters in certain tumors predicts the likelihood of clinical response to treatment [53,54].

Considering the methylome coverage requirements, sample throughput, and resources available, the current range of technologies is able to meet most needs. Over the last few years, a considerable increase in the stability, throughput, and quality of NGS has been attained. These massive parallel sequencing technologies allow for comprehensive interrogation of genomes without prior knowledge of sequence or annotation. The relative low amounts of starting material reduced the errors and bias caused by sample preparation and amplification [63]. Next-generation reads are generated from fragmented and adapter-ligated DNA libraries that have never been subjected to conventional vector-based cloning, enabling to circumvent some of the sequencing bias of cloned DNA sequences that affect genome identification in sequencing projects [16]. Furthermore, high sensitivity, specificity, and scalability make this technology a powerful tool for the search of novel epigenetic biomarkers [9]. Additionally, signal quantification from sequence-based approaches focus on counting sequence tags rather than relative measures between samples, enabling an unlimited, fully-quantitative result [11]. Lastly, the increase in the amount of data generated per run and the decrease in reagent costs resulted in a higher cost-effectiveness of NGS for methylome profiling [63].

Currently, NGS is used to characterize several types of cancer and enabled the construction of large-scale databases such as The Cancer Genome Atlas (TCGA) (http://cancergenome.nih.gov/) and International Cancer Genome Consortium (http://icgc.org). These databases contain hundreds of cancer profiles based on whole-genome sequencing, gene expression and protein profiling, RNA sequencing, methylome analysis and copy number variation [50].

However, to best suit clinical purposes, targeted NGS panels are now emerging, assessing specific gene mutations that may assist in diagnosis and selection of targeted therapy. With targeted sequencing, a subset of genomic regions are isolated and sequenced, allowing researchers to focus time, costs, and data analysis on specific areas of interest and enabling sequencing at much higher coverage levels (500–1000× higher). For assay development, amplicon-based methods have been preferentially used because of their simplified workflow and small amounts of input DNA required. However, capture sequencing has emerged as an alternative approach because of high testing accuracy with respect to sequencing complexity and uniformity of coverage [64].

The main drawback for NGS in the clinical setting is the infrastructure required, including computer capacity and storage, and, importantly, expert bioinformatician support to comprehensively analyze and interpret the vast amount of data that is generated [13]. Moreover, the effective interpretation of datasets generated in different laboratories with common DNA profiling techniques requires the adoption of standards in both experimental and computational methods to allow for meaningful comparisons between experiments [10]. Importantly, quality control matrices and procedures should be adopted during base resolution methylome profiling. Methylation sequencing quality control includes reads alignment, methylation scoring, methylation heterogeneity assessment, genomic features annotation, data visualization, and determination of differentially methylated cytosines. These quality control steps are integrated into analytic pipelines, such as MethylQA or BSeQC tools [65,66].

## 4. Non-Invasive DNA Methylation Detection Using Next-Generation Sequencing: Technical Advances and Challenges

Until a few years ago, the majority of DNA methylation studies investigated genome methylation status using tissue-extracted DNA. However, because the tissue availability depends on invasive procedures, it limits it usefulness and, especially in biopsies, heterogeneity might be underestimated due to sampling limitations. Heterogeneous methylation found in tissues makes the fraction of methylation segments difficult to quantify with precision, compromising its use as a biomarker [67].

Currently, sequencing methylated DNA in a single-cell is now possible using a variety of experimental approaches, providing provides further insights into the links between cell’s phenotype and genotype. Parallel measurement of single-cell epigenomes might improve our understanding of normal developmental and disease processes [68]. As previously mentioned, DNA methylation commonly correlates with gene expression variations in mammals. Thus, to explore the cause-consequence basis for this association, measurements of both DNA methylation and transcript expression for the same single-cells is mandatory to uncover the dynamics of heterogeneous cell populations [69]. Currently several methods, including methylome and transcriptome sequencing from single-cell (scM&T-seq) and single-cell triple omics sequencing (scTrio-seq), allow for the consideration of whether DNA methylation extent of different functional elements in the genome impacts on the expression levels of genes in single cells [70,71,72].

Researchers are now investing in the development of less invasive and more accessible approaches to complement, and eventually substitute, tissue DNA analysis. Bodily fluids, such as plasma, serum, urine, or even saliva, often harbor increased cell-free DNA levels, which may be potentially detected using massive parallel sequencing [73]. Thus, NGS constitutes a promising tool for clinical biomarkers’ research due to its high sensitive and time/cost efficiency [74]. The use of NGS in liquid biopsies showed great potential for molecular testing and currently this methodology is largely applied for clinically relevance hotspot mutations detection. However, these types of genetic mutations are only detected in a small subset of patients, limiting its use for early diagnosis strategies [75,76,77]. With the emergence of epigenetics, aberrant DNA methylation profiles were shown to be early and common events during illness development, enabling a more robust detection and higher sensitivity in diagnosis [1,78]. Thus, escalating liquid biopsies molecular testing using NGS technologies to identify epigenetic signatures will potentially increase the diagnostic yield and clinical usefulness [79]. Furthermore, different tissue types may share similar genotypes but display different methylation profiles, and, thus, type-specific methylation signatures can potentially be used to identify the tissue of origin [80,81].

Regarding applications to clinical practice, NGS has been used to determine genome-wide profiles in serum or plasma from different cancers [82,83,84]. The single base resolution is attractive because it allows for precise mapping of relevant disease-specific sites. However, the combination of high cost of sequencing entire genomes and the large number of samples needed to provide adequate statistical power makes WGBS not economically viable as a screening tool for disease association studies at this time. Currently, a commonly used cost-effective alternative is methylation-capture followed by NGS [85]. Nevertheless, the application of these protocols to the methylation fraction of cell-free DNA still requires overcoming several technical challenges. Firstly, cell-free DNA occurs at a very low concentration levels leading to non-specific bindings during sample capture process and constitutes a critical limitation for this methodology. A second drawback results from the methylation enrichment step, which recovers a minimal fraction of the total DNA input, reducing the amount of DNA available for NGS library preparation [86,87]. Furthermore, ameliorate the quality of cell-free DNA extraction procedures, improving target candidate’s selection to increase sequencing coverage and find suitable reference DNA methylomes might speed-up the use of NGS technologies in non-invasive epigenetic tests to assist in diagnosis and prognostication.

## 5. Conclusions

The epigenetic community rapidly combined NGS with established DNA methylation capture methods enabling the improvement of the methylome analysis. Hence, in the last years, NGS became an effective tool for DNA methylation profiling at a single-base level and at relatively affordable price. Furthermore, the use of NGS contributed to increased knowledge on differentially methylated DNA regions and the discovery of new gene regulatory elements involved in the epigenetic machinery. These massive-parallel technologies offer great promise for decoding the nature and patterns of DNA modifications, as well as their implications in the various pathologic and physiologic processes. There are, however, other applications of high-throughput sequencing technologies in base modification. Information about DNA methylation patterns and distribution in the human genome is also important for personalized epigenomic-based therapy development.

## Figures and Tables

**Table 1:** Comparison of key features of the different genome-wide approaches for DNA methylation profiling. CpGs: Cytosine-phosphate-Guanine; bp: base pair.

	Affinity Enrichment-Based Methods	Restriction Enzymes-Based Methods	Bisulfite Conversion-Based Methods
**Resolution**	~150 bp	Single-base	Single-base
**Reads/sample**	~30–50 million reads	~10 million reads	>500 million reads
**CpGs covered**	~23 million CpGs	~2 million CpGs	>28 million CpGs
**Pros**	Cost-effective method No mutations introduced	High sensitivity with lower costs	Evaluate methylation status of every CpG site
**Cons**	Biased toward hypermethylated regions Inability to predict absolute methylation level	CpGs in regions without the enzyme restriction site are not covered	Higher costs Requires high DNA input Substantial DNA degradation after bisulfite treatment
**Application**	Suitable for rapid, large scale and low-resolution studies	Suitable for site-specific/targeted studies	Suitable for high resolution studies

**Table 2:** Comparison of characteristics of the main next-generation sequencing technologies.

Sequencing Platform Developers	Sequencing Principle	Key Features	Limitations	Reference
**Illumina**	Sequencing by synthesis	High throughput	Higher cost per read	[55,56,57]
**Life Technologies Ion Torrent**	Polymerization	Simple detection method	Low read number per run	[58,59]
**Pacific Biosciences PacBio**	Single molecule real time ligation	Single molecule detection and long read length	High error rates (13%) and low read number per run	[60]
**Oxford Nanopore**	Nanopore sensing	Single molecule and label-free detection with reduced costs	High error rates (38.2%)	[61,62]
